# Meteorological Report for October

**Published:** 1880-11

**Authors:** 


### METEOROLOGICAL REPORT FOR OCTOBER, 1880.

| RAIN. | | Mean daily Barometer. | Mean daily Thermometer | Mean daily Humidity. | Maximum Thermometer | Minimum Thermometer | Prevailing direction of Wind. | Maximum hourly velocity of Wind, (Daily.) |
|---|---|---|---|---|---|---|---|---|
| DATE | IN. | | | | | | | |
| 1 | 14 | 30.241 | 60.0 | 51.7 | 68 | 47 | E. | ......... |
| 2 | 05 | 30.188 | 62.7 | 54.0 | 71 | 50 | N. E. | ......... |
| 3 | 0 | 30.115 | 65.5 | 58.0 | 74 | 53 | E. | ......... |
| 4 | 0 | 29.977 | 68 0 | 82.7 | 77 | 63 | E. | ......... |
| 5 | 25 | 29.948 | 62.2 | 85.3 | 72 | 64 | N. W. | ......... |
| 6 | 03 | 29.959 | 67.7 | 74.0 | 76 | 58 | N. W. | ......... |
| 7 | 32 | 30 031 | 59.5 | 86.3 | 66 | 55 | N. E. | ......... |
| 8 | 32 | 30 000 | 58.2 | 77.7 | 61 | 64 | N. E. | ......... |
| 9 | 0 | 30.085 | 63.2 | 74.0 | 69 | 55 | E. | ......... |
| 10 | 0 | 30.161 | 67.0 | 71.3 | 73 | 62 | E. | ......... |
| 11 | 37 | 30 206 | 69 2 | 75.0 | 75 | 63 | E. | ......... |
| 12 | 18 | 30.126 | 70.7 | 77.3 | 79 | 64 | N. W. | ......... |
| 13 | 1.01 | 30.148 | 67.7 | 62.7 | 76 | 64 | E. | ......... |
| 14 | 0 | 30.154 | 63 0 | 71.0 | 69 | 57 | E. | ......... |
| 15 | 0 | 29.998 | 68 7 | 80.3 | 77 | 60 | S. E. | ......... |
| 16 | 0 | 29.918 | 63.2 | 76.3 | 73 | 57 | N. W. | ......... |
| 17 | 0 | 30 173 | 52.5 | 47.0 | 59 | 49 | N. | ......... |
| 18 | 0 | 30.397 | 51.0 | 44.3 | 56 | 42 | N. W. | ......... |
| 19 | 0 | 30.347 | 56.7 | 56.7 | 65 | 44 | S. E. | ......... |
| 20 | 0 | 30.245 | 61.5 | 55 0 | 68 | 50 | S. E. | ......... |
| 21 | 0 | 30.127 | 56.0 | 72.3 | 60 | 52 | E. | ......... |
| 22 | 0 | 29.918 | 56.7 | 65.0 | 66 | 51 | N. W. | ......... |
| 23 | 0 | 30.128 | 50.2 | 50.7 | 56 | 43 | N. W. | ......... |
| 24 | 3.17 | 30.310 | 55.5 | 42.7 | 62 | 43 | N. W. | ......... |
| 25 | 13 | 30.256 | 54.5 | 42 0 | 64 | 41 | E. | ......... |
| 26 | 0 | 30.001 | 59.5 | 47.7 | 67 | 50 | S. W. | ......... |
| 27 | 15 | 29.959 | 63.0 | 66.3 | 70 | 52 | S. E. | ......... |
| 28 | 0 | 29.964 | 62.2 | 88.7 | 67 | 59 | E. | ......... |
| 29 | 0 | 29 793 | 59.2 | 91.3 | 62 | 57 | N. E. | ......... |
| 30 | 0 | 29.729 | 62.2 | 83.0 | 66 | 59 | N. W. | ......... |
| | | 29.979 | 50.7 | 65 3 | 62 | 47 | N. W. | ......... |
| Sums | | 30 083 | 61.0 | 66.9 | 67.9 | 53.6 | ......... | ......... |

Mean Monthly Barometer, 30 083.
Highest Barometer, 30.452, on the 18th, 10:30 A.M.
Lowest Barometer, 29.680, on the 30th, 2:30 A.M.
Monthly range of Barometer, 0.772.
Highest Temp. 79°. Lowest Temp. 41°.
Monthly range of Temperature, 38°.
Greatest daily range of Temperature, 23°.
Least daily range of Temperature, 5°.
Mean of Maximum Temperatures, 67°.9.
Mean of Minimum Temperatures, 53°.6.
Mean daily range of Temperature, 14°3.
Total rainfall or melted snow, 2 08 inches.
Prevailing wind, East. Total movement of wind, 56.65 miles.
Maximum velocity of wind and direction, 23 miles per hour; N.W., o 1 31st.
Number of fair days, 9.
Number of cloudy days on which rain or snow fell, 8.
Number of cloudy days on which no rain or snow fell, 8.
Total number of days on which rain fell, 18.

H. HALL, Corp'l. Signal Service U.S. A

Atlanta, Ga., October, 1880.

